# Epigenetic treatment of synaptic and behavioral deficits in *Dyrk1a*-mutant mice

**DOI:** 10.1038/s41386-026-02353-x

**Published:** 2026-01-20

**Authors:** Chih-Hung Lin, Mingjun Yu, Prachetas Jai Patel, Pei Li, Zhen Yan

**Affiliations:** Department of Physiology and Biophysics, Jacobs School of Medicine and Biomedical Sciences, State University of New York at Buffalo, Buffalo, NY USA

**Keywords:** Autism spectrum disorders, Epigenetics and plasticity

## Abstract

Haploinsufficiency of *Dyrk1a*, which encodes the dual-specificity tyrosine phosphorylation regulated kinase 1A (DYRK1A), has been causally linked to autism. Here we examined transcriptomic, electrophysiological and behavioral alterations in mice carrying a loss-of-function mutation of *Dyrk1a* (*Dyrk*^mut^). We found that genes downregulated in prefrontal cortex (PFC) of male and female *Dyrk*^mut^ mice were enriched in chemical synaptic transmission and trans-synaptic signaling. In PFC pyramidal neurons of *Dyrk*^mut^ mice, the frequency of synaptic-driven spontaneous action potentials (sAP) was significantly reduced, and glutamatergic excitatory postsynaptic currents (EPSC) and GABAergic inhibitory postsynaptic currents (IPSC) were markedly diminished. Furthermore, autism-like social preference deficits and elevated anxiety were manifested in *Dyrk*^mut^ mice of both sexes. A short treatment of *Dyrk*^mut^ mice with an inhibitor of the epigenetic corepressor lysine-specific histone demethylase 1A (LSD1) led to a significant elevation of sAP frequency, EPSC and IPSC in PFC pyramidal neurons of *Dyrk*^mut^ mice. Moreover, the LSD1 inhibitor ameliorated social deficits in *Dyrk*^mut^ mice and reduced anxiety in *Dyrk*^mut^ males. Therefore, these data have not only revealed synaptic and behavioral deficits in PFC induced by *Dyrk1a* mutation, but also uncovered the therapeutic potential of LSD1 inhibition in *Dyrk1a*-deficient conditions.

## Introduction

DYRK1A (dual-specificity tyrosine phosphorylation regulated kinase 1A), which is encoded by the *DYRK1A* gene on human chromosome 21, plays a pivotal role in diverse cellular and molecular processes, such as transcriptional regulation, neurogenesis, apoptosis, and synaptic plasticity, from early embryogenesis through late aging [1–4]. By phosphorylating and interacting with histone acetyl transferase p300 and CBP, DYRK1A could regulate enhancer activity and gene expression [5]. As a negative regulator of the G1-S phase transition, DYRK1A overexpression inhibits proliferation and induces premature neuronal differentiation [6]. By phosphorylating caspase 9 to inhibit its activity, DYRK1A could regulate apoptosis [7]. Furthermore, DYRK1A is involved in synaptic plasticity through its phosphorylation of the transcription factors NFAT and CREB [8, 9]. DYRK1A also contributes to neurodegenerative disorders including Alzheimer’s disease and Parkinson’s disease by phosphorylating and regulating Mapt/Tau mRNA splicing [10], α-synuclein [11] and parkin [12].

With a crucial role in the brain, *Dyrk1a* dosage determines the state of physiology and pathology [2, 13, 14]. DYRK1A overexpression leads to Down syndrome [15], while DYRK1A haploinsufficiency causes autism [16, 17]. Most of DYRK1A missense mutations result in enzymatically dead DYRK1A proteins [18]. Mice with *Dyrk1a* deletion had the decreased brain mass and neuronal size, as well as structural hypoconnectivity [19, 20], which is consistent with the role of *Dyrk1a* in postembryonic neurogenesis in Drosophila [21]. Deregulation of synaptic proteins was found with *Dyrk1a* gene copy number variations in glutamatergic neurons [14], and synaptic F-actin was significantly decreased following *Dyrk1a* knockdown [22]. *Dyrk1a*^*+/−*^ mice exhibited the impaired hippocampal inhibitory circuit and the reduced excitatory synaptic currents [23, 24]. Behavioral abnormalities, such as impaired long-term explicit memory, reduced social interaction and recognition, and increased susceptibility to epilepsy, were also found in *Dyrk1a* haploinsufficient mice [14, 18, 23–25]. These phenotypes are consistent with the clinical core phenotypes of patients with *Dyrk1a* haploinsufficiency, including microcephaly, developmental delay, seizures, and autism [26–30].

One key issue is to find effective treatment for diseases linked to *Dyrk1a* haploinsufficiency. Here, using a mouse model carrying mutated *Dyrk1a, Dyrk*^mut^ [31], we demonstrated the alteration of synaptic genes and synaptic functions in prefrontal cortex, as well as the manifestation of autism-related behaviors. Our previous study found that a specific inhibitor of the epigenetic corepressor lysine-specific histone demethylase 1A (LSD1) had promising effects in treating *Shank3*-deficient autism model [32], which prompted us to test its therapeutic potential in *Dryk1a* mutant mice. We found that a short treatment of adult *Dyrk*^mut^ mice with an LSD1 inhibitor led to the significant rescue of synaptic and behavioral deficits. It supports the concept of targeting epigenetic enzymes for autism treatment [32–38].

## Methods

### Animals and chemicals

All animal experiments followed the protocol approved by the Institutional Animal Care and Use Committee (IACUC) of the State University of New York (SUNY) at Buffalo, and were in compliance with US National Research Council’s Guide for the Care and Use of Laboratory Animals. *Dyrk*^mut^ mice (Strain # 041838-JAX in Jackson Lab) contained a trap mutation with a beta-geo cassette insertion into intron 4 [31]. Heterozygous *Dyrk*^mut^ mice were used in this study. They were maintained by crossing to (B6x129)F1 mice. Mice were group-housed under the controlled conditions of temperature (22 °C), humidity (56%), and a 12/12 h light/dark cycle, with food and water *ad libitum*.

GSK-LSD1 (Tocris, Cat. # 5361) was dissolved in double-distilled water as a stock solution, and then aliquoted and stored at −20 °C. The stock solution was diluted with sterile saline as a working solution before experiments. Mice (2–4 months old) were injected intraperitoneally with sterile saline (vehicle control) or GSK-LSD1 (5 mg/kg) once daily for 3 consecutive days. Mice were utilized for various experiments within 1–8 days post-injection.

### RNA-sequencing (RNA-seq) and bioinformatic analysis

After animal sacrificing, PFC regions were collected, total RNA was isolated using the RNAeasy Mini kit (Qiagen), coupled to an RNase-free DNase step (Qiagen). RNA sequencing libraries were constructed by TruSeq stranded total RNA plus Ribo-zero kits (Illumina). Sequencing was carried out with the HiSeq 2500 platform (Illumina). RNA-seq tags reads were aligned to the mouse reference genome mm10 using RNA STAR (Galaxy version 2.7.5b). Gene expression of mapped reads was then measured using featureCounts (Galaxy Version 1.6.4+galaxy1). Genes without a canonical name, as well as ribosomal and mitochondrial RNA products, were excluded. The first batch of RNA-seq was performed using PFC from 3 pairs of female Wild-type (WT) and *Dyrk*^mut^ mice. The second batch of RNA-seq was performed using PFC from 3 pairs of different female WT and *Dyrk*^mut^ mice, and 3 pairs of male WT and *Dyrk*^mut^ mice.

Differentially expressed genes (DEGs), obtained using DEseq2 (Galaxy Version 2.11.40.2), were defined with at least 1.1 Fold Change (FC) and *p* value ≤ 0.05. Enrichment analyses of DEGs were conducted using EnrichR [https://maayanlab.cloud/Enrichr/]. String database [https://string.db] was used to create protein-protein interaction (PPI) network for genes in top enriched categories. InteractiveVenn [http://www.interactivenn.net/] was used to detect overlapping genes among gene sets. Heatmap of normalized gene expression was generated using Phantasus [https://ctlab.itmo.ru/phantasus].

### Electrophysiological measurements

Slice recording of action potential firings and synaptic currents in layer V pyramidal neurons of medial prefrontal cortex (mPFC) from mice (2–4 months old) used whole-cell current-clamp or voltage-clamp as what we previously illustrated [32, 35, 39–41]. In brief, mice under isoflurane-induced anesthesia were decapitated by the guillotine, and brains were taken out and put in an icy sucrose solution (in mM: 234 sucrose, 15 HEPES, 11 glucose, 4 MgSO_4_, 2.5 KCl, 1 Na_2_HPO_4_, 0.1 CaCl_2_, pH 7.35, 300 mOsm), and sliced into 300 µm coronal slices with a vibratome (Leica VT1000). The brain slices were recovered by incubating at 32 °C oxygenated (95% O_2_/5% CO_2_) artificial cerebrospinal fluid (aCSF) (in mM: 130 NaCl, 26 NaHCO_3_, 10 glucose, 3 KCl, 1.25 NaH_2_PO_4_, 1 CaCl_2_, 5 MgCl_2_, pH 7.4, 300 mOsm) for 1 h and thereafter at the room temperature (22–24 °C).

After recovery, the brain slice was submerged in continuously flowing oxygenated aCSF in the perfusion chamber of the stage of the upright microscope (Olympus BX51WI). With the use of Multiclamp 700A amplifier, Digidata 1322A data acquisition system, and Clampex software (Molecular Devices, Sunnyvale, CA), neurons were visualized with a 40× water-immersion lens and recorded. Recording electrodes were pulled by the borosilicate glass capillaries (1.5/0.86 mm OD/ID) via a micropipette puller (Model P-97, Sutter Instrument Co., Novato, CA). The resistance of the electrode was 2–4 MΩ, and the threshold value of series resistance is 25 MΩ.

During recording, slices were perfused with modified aCSF (in mM: 130 NaCl, 26 NaHCO_3_, 10 glucose, 3.5 KCl, 1.25 NaH_2_PO_4_, 1 CaCl_2_, 0.5 MgCl_2_, pH 7.4, 300 mOsm). To record synaptic-driven spontaneous action potentials (sAP), a small (<50 pA) adjusting current (positive DC) was injected to elevate the membrane potential (inter-spike potential: −60 mV) [42, 43]. Recording pipettes were filled with the internal solution (in mM: 124 K-gluconate, 10 HEPES, 6 KCl, 5 EGTA, 1 MgCl_2_, 0.5 CaCl_2_, 22 phosphocreatine, 2 Na_2_ATP, 0.2 Na_2_GTP, pH 7.3, 270 mOsm). To record synaptic currents, the internal solution contained (in mM: 130 Cs-methanesulfonate, 10 CsCl, 10 HEPES, 5 EGTA, 4 NaCl, 1 MgCl_2_, 16 phosphocreatine, 5 MgATP, 0.5 Na_2_GTP, 2 QX-314, pH 7.3, 270 mOsm). Neurons were held at −70 mV for excitatory postsynaptic currents (EPSC) and 0 mV for inhibitory postsynaptic currents (IPSC) recordings. A bipolar stimulating electrode (FHC, Bowdoinham, ME) was positioned ~100 μm away. A S48 pulse generator (Grass Technologies, West Warwick, RI) transferred electrical pulses (0.058 ms, 100 μA) to the neuron to evoke synaptic currents.

### Behavioral assays

All behavior tests were conducted under dim light from 12 p.m. to 6 p.m. Mice (2–3 months old) were transferred to the behavior testing room for at least 1 h before behavioral assays. A 24-h interval was maintained between different tests to prevent potential interference (Elevated plus maze test on day 1, Social preference test on day 2). Testing apparatus was cleaned with 70% ethanol to avoid interference between different animals. And male and female mice are tested separately. Any-maze software (Stoelting Co.) was used to track, record and measure automatically.

#### Social preference test

As described previously [44, 45], a three-chamber apparatus (H: 20 inches L: 41.75 inches, W: 22 inches) with two inverted pencil cups placed in the side chambers (each topped with a glass bottle) was used. During habituation, animals were put in the middle chamber and allowed to explore the 3-chamber apparatus with two empty cups for 10 min. In the 1st test phase, two identical paper balls were placed underneath the cups, and mice were put in the apparatus to explore for 10 min. Mice were then placed in the holding cages for 5 min to rest. In the 2nd test phase, one social stimulus (a sex- and age-matched grouped-housed stranger WT mouse) and one non-social stimulus (a wooden block) were placed underneath the cups, and mice were put in the apparatus to explore for 10 min. The time spent interacting with social (S) and non-social (NS) stimuli was recorded. Social preference index was calculated as (T_S_ − T_NS_)/ (T_S_ + T_NS_) to evaluate the sociability. Total distance traveled was measured to evaluate the locomotor activity.

#### Elevated plus maze (EPM) test

As previously described [40, 46], the plus shaped EPM (19.88 inches above the floor) consisted of two open arms (L:14 inches, W: 3 inches) and two closed arms (L:14 inches, W: 3 inches; H: 6.5 inches, with black walls). Mice were placed in the center area of EPM and headed to the same open arm. The time spent in open and closed arms, and total distance traveled were recorded during the 10-min test. Each mouse was tested only once, because repeated EPM tests significantly affected mouse behaviors.

### Western blotting

Nuclear extraction from mouse brain was performed as we described before [46] with modifications. Briefly, the PFC tissue was homogenized with 250 μl hypotonic buffer (20 mM Tris-HCl, pH 7.4, 10 mM NaCl, and 3 mM MgCl_2_) including protease inhibitor cocktail (1:50), 1 mM PMSF and 10% NP-40. Homogenization was facilitated by passing the lysate through a 1 ml syringe fitted with a 26-gauge needle (15 strokes). After incubation on ice for 30 min, 12.5 μl NP-40 (10%) was added to the homogenate, followed by vortexing for 10 s. Then the homogenate was centrifuged at 3000 × *g* at 4 °C for 10 min. The nuclear pellet was resuspended in 1% SDS with protease inhibitor cocktail and 1 mM PMSF. Protein concentration was measured by Pierce™ BCA Protein Assay Kit (Thermo Scientific, 23227). All the samples were set to the same concentration and boiled with 4× SDS loading buffer for 10 min. According to the size of the proteins, 12% SDS-polyacrylamide gels were used. Samples (3–10 μg) were loaded according to the sensitivity of different antibodies. Western blotting experiments for nuclear proteins were performed with antibodies against Histone 3 (cell signaling, 4499, 1:1000), H3K4me2 (cell signaling, 9725, 1:1000), and H3K9me2 (cell signaling, 4658, 1:1000). Blots were incubated with the HRP-conjugated second antibodies (1:2000) for 2 h at room temperature. SuperSignal™ West Pico PLUS Chemiluminescent Substrate (ThermoFisher Scientific, 34577) was used to detect protein signals.

### Statistical analysis

Data analyses were performed by MiniAnalysis (Synaptosoft, NJ), Clampfit (Molecular Devices, Sunnyvale, CA), and GraphPad Prism (GraphPad). Significant differences between two groups were compared by Student’s *t* test. For groups with significantly different variances, Welch’s *t* test was used. For groups containing data without normal distribution, Mann–Whitney (M-W) test was used. Significant differences among more than two groups were analyzed with one-way ANOVA followed by Tukey’s multiple comparisons. All data were shown as mean ± SEM.

## Results

### RNA sequencing reveals the downregulation of synaptic genes in *Dyrk1a*^mut^ mice

**Fig. 1:**
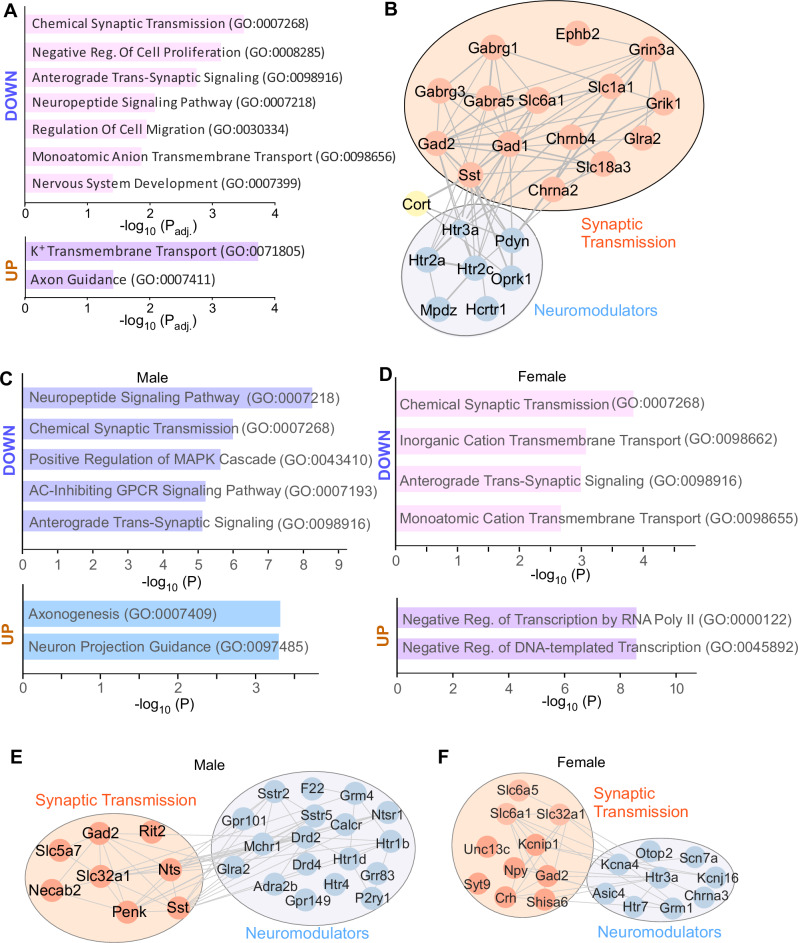
*Dyrk1a* mutation leads to the downregulation of synaptic genes. **A** Gene Ontology (GO) analysis of downregulated (DOWN) and upregulated (UP) genes in female *Dyrk1a*^mut^ mice, compared to female WT mice. **B** Protein-protein interaction (PPI) network of downregulated genes in top GO categories of female *Dyrk1a*^mut^ mice. GO analysis of DOWN and UP DEGs in another batch of male (**C**) and female (**D**) *Dyrk1a*^mut^ mice, compared to sex-matched WT mice. PPI network of downregulated genes in top GO categories of male (**E**) and female (**F**) *Dyrk1a*^mut^ mice.

To find out genome-wide gene expression alterations by *Dyrk1a* mutation, we performed RNA sequencing (RNA-seq) in prefrontal cortex (PFC) of female WT vs. *Dyrk1a*^mut^ mice. We found 1056 differentially expressed genes (DEGs), with 544 genes downregulated and 511 genes upregulated (Supplementary Table 1). Gene Ontology (GO) analysis of DEGs by *Dyrk1a* mutation (Fig. 1A) uncovered that the downregulated (DOWN) genes were enriched in chemical synaptic transmission, trans-synaptic signaling, and neuropeptide signaling pathway, while the upregulated (UP) genes were enriched in potassium ion transmembrane transport and axon guidance.

Protein-protein interaction (PPI) networks of the downregulated genes in top GO categories of female *Dyrk1a*^mut^ mice (Fig. 1B) demonstrated two highly-connected clusters: synaptic transmission genes including GABA synthesis enzymes (*Gad1* and *Gad2*), GABA transporter (*Slc6a1*), GABAergic neuronal marker (*Sst*), GABA_A_ receptors (*Gabrg1*, *Gabrg3*, and *Gabra5*), glutamate receptors (*Grin3a* and *Grik1*) and glutamate transporter (*Slc1a1*); and neuromodulator genes including serotonin receptors (*Htr2a*, *Htr2c*, *Htr3a*), orexin receptor (*Hcrtr1*), opioid peptide and receptor (*Pdyn* and *Oprk1*).

To confirm these results, we performed additional RNAseq experiments with PFC from another batch of male and female WT vs. *Dyrk1a*^mut^ mice (Supplementary Tables 2, 3). GO analysis (Fig. 1C, D) found that downregulated protein-coding genes by *Dyrk1a* mutation (355 in males, 380 in females) were both enriched in chemical synaptic transmission and trans-synaptic signaling. Upregulated protein-coding genes by *Dyrk1a* mutation (275 in males, 631 in females) were enriched in different pathways: axonogenesis for males and transcriptional regulation for females.

PPI networks of the downregulated genes in top GO categories of male *Dyrk1a*^mut^ mice (Fig. 1E) contained two highly-connected clusters: synaptic transmission genes including GABA synthesis enzyme (*Gad2*), vesicular GABA transporter (*Slc32a1*) and GABAergic neuronal marker (*Sst*); and neuromodulator genes including serotonin receptors (*Htr1b*, *Htr1d*, *Htr4)* and dopamine receptors (*Drd2, Drd4*). PPI of downregulated genes in top GO categories from the second batch of female *Dyrk1a*^mut^ mice (Fig. 1F) also contained synaptic transmission genes (*Gad2, Syt9, Slc6a1, Slc32a1*) and neuromodulator genes (*Htr3a, Htr7, Grm1*). Significant overlap of downregulated genes in the different batches of RNAseq data from male and female *Dyrk1a*^mut^ mice were identified (Supplementary Table 4, over-enriched: 3.34–5.28 fold, hypergeometric *p*-value: 7.9e−09 to 1.7e−30), and these commonly downregulated genes were enriched in chemical synaptic transmission, neuropeptide signaling pathway, and trans-synaptic signaling. These data suggest that Dyrk1a may play an important role in synaptic regulation.

### Electrophysiological recordings demonstrate the loss of excitatory and inhibitory synaptic transmission in PFC of *Dyrk1a*^mut^ mice

**Fig. 2:**
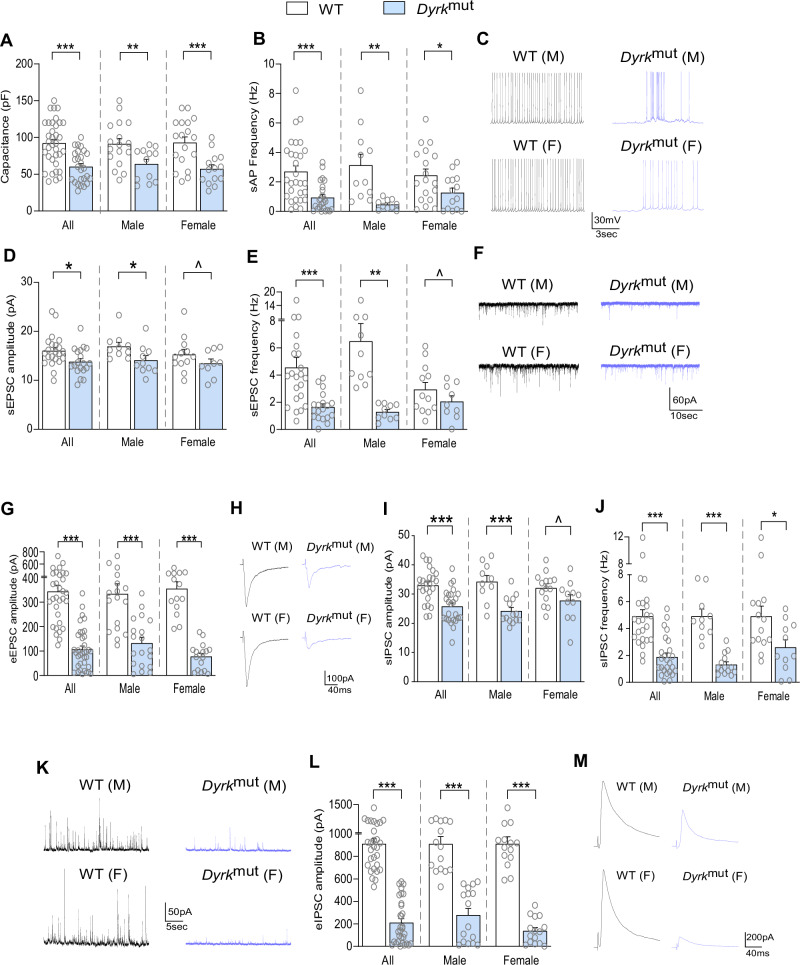
*Dyrk1a*^mut^ mice exhibit the reduced excitability and diminished glutamatergic and GABAergic synaptic transmission in PFC pyramidal neurons. **A** Bar graphs of membrane capacitance of PFC pyramidal neurons from male and female WT vs. *Dyrk1a*^mut^ mice. **B** Bar graphs of spontaneous action potential (sAP) frequency of PFC pyramidal neurons from male and female WT vs. *Dyrk1a*^mut^ mice. **C** Representative sAP traces. Bar graphs of sEPSC amplitude (**D**) or frequency (**E**) in PFC pyramidal neurons from male and female WT vs. *Dyrk1a*^mut^ mice. **F** Representative sEPSC traces. **G** Bar graphs of eEPSC amplitude in PFC pyramidal neurons from male and female WT vs. *Dyrk1a*^mut^ mice. **H** Representative eEPSC traces. Bar graphs of sIPSC amplitude (**I**) or frequency (**J**) in PFC pyramidal neurons from male and female WT vs. *Dyrk1a*^mut^ mice. **K** Representative sIPSC traces. **L** Bar graphs of eIPSC amplitude in PFC pyramidal neurons from male and female WT vs. *Dyrk1a*^mut^ mice. **M** Representative eIPSC traces. All data are shown as mean ± SEM, ^*p* < 0.2, *p < 0.05, **p < 0.01, ***p < 0.001, unpaired *t*-test.

With the downregulation of synaptic genes in *Dyrk1a*^mut^ mice by RNA-seq, we next examined the changes in synaptic function by *Dyrk1a* mutation using whole-cell patch-clamp recordings. Layer V medial PFC (mPFC) pyramidal neurons were selected because these cells exhibit the clearest deficits in the brains of children with autism [47]. Neuronal sizes were significantly smaller in *Dyrk1a*^mut^ mice than WT counterparts, as demonstrated by the significantly lower membrane capacitance (Fig. 2A, *n* = 12–18 cells/3–5 mice of each sex per group; all: 34.6% reduction, *t*_59_ = 4.7, *p* < 0.0001; male: 30.0% reduction, *t*_27_ = 2.8, *p* = 0.0095; female: 38.6% reduction, *t*_29_ = 3.8, *p* = 0.0007, *t*-test).

We first measured the synaptic-driven spontaneous action potential (sAP), and found that sAP frequency was significantly lower in PFC pyramidal neurons from *Dyrk1a*^mut^ mice, compared to those from WT mice, and the loss was particularly strong in *Dyrk1a*^mut^ males (Fig. 2B, C, *n* = 10–18 cells/2–5 mice of each sex per group; all: 65.5% reduction, *t*_42_ = 4.1, *p* = 0.0002; male: 85.1% reduction, *t*_10_ = 3.6, *p* = 0.0049; female: 48.1% reduction, *t*_29_ = 2.2, *p* = 0.034, *t*-test).

The lower sAP frequency in *Dyrk1a*^mut^ mice suggests the loss of synaptic activity. To test this, we measured spontaneous excitatory postsynaptic currents (sEPSC). We found that male *Dyrk1a*^mut^ mice had significantly reduced sEPSC amplitude and frequency, while female *Dyrk1a*^mut^ mice had only a trend of reduction (Fig. 2D–F, *n* = 9–12 cells/3–5 mice of each sex per group; sEPSC amplitude, all: 14.0% reduction, *t*_39_ = 2.4, *p* = 0.023; male: 16.7% reduction, *t*_18_ = 2.3, *p* = 0.036; female: 12.0% reduction, *t*_19_ = 1.3, *p* = 0.20; sEPSC frequency, all: 63.7% reduction, *t*_25_ = 3.7, *p* = 0.0010; male: 80.1% reduction, *t*_9_ = 4.0, *p* = 0.003; female: 29.9% reduction, *t*_19_ = 1.3, *p* = 0.21, *t*-test). Next, we recorded EPSC evoked by electrical stimulations (eEPSC). We found that both sexes of *Dyrk1a*^mut^ mice exhibited significantly diminished eEPSC amplitudes (Fig. 2G, H, *n* = 14–19 cells/4–5 mice of each sex per group; all: 69.0% reduction, *t*_47_ = 8.1, *p* < 0.0001, male: 60.4% reduction, *t*_25_ = 4.3, *p* = 0.0002; female: 78.1% reduction, *t*_18_ = 8.9, *p* < 0.0001; *t*-test).

Given the downregulation of GABA synapse genes in *Dyrk1a*^mut^ mice by RNA-seq, we further measured GABAergic synaptic currents in layer V mPFC pyramidal neurons. Compared to WT mice, the amplitude and frequency of spontaneous inhibitory postsynaptic currents (sIPSC) were significantly reduced in male *Dyrk1a*^mut^ mice, while only the frequency of sIPSC was significantly lowered in female *Dyrk1a*^mut^ mice (Fig. 2I–K, *n* = 10–14 cells/3–4 mice of each sex per group; sIPSC amplitude, all: 21.8% reduction, *t*_47_ = 4.3, *p* < 0.0001; male: 29.3% reduction, *t*_15_ = 4.2, *p* = 0.0008; female: 13.4% reduction, *t*_19_ = 1.7, *p* = 0.10; sIPSC frequency, all: 61.6% reduction, *t*_38_ = 5.3, *p* < 0.0001; male: 73.1% reduction, *t*_12_ = 6.3, *p* < 0.0001; female: 47.0% reduction, *t*_22_ = 2.4, *p* = 0.023, *t*-test). Both sexes of *Dyrk1a*^mut^ mice exhibited the significantly diminished amplitude of IPSC evoked by electrical stimulations (eIPSC) (Figs. 2L and 2M, *n* = 14–16 cells/4–5 mice of each sex per group; all: 76.9% reduction, *t*_54_ = 12.4, *p* < 0.0001; male: 69.4% reduction, *t*_28_ = 7.2, *p* < 0.0001; female: 84.9% reduction, *t*_19_ = 11.4, *p* < 0.0001, *t*-test). Altogether, these electrophysiological results demonstrate that both excitatory and inhibitory synaptic transmission in mPFC are diminished by *Dyrk1a* mutation.

### Behavioral assays uncover the social deficits and elevated anxiety in *Dyrk1a*^mut^ mice

**Fig. 3:**
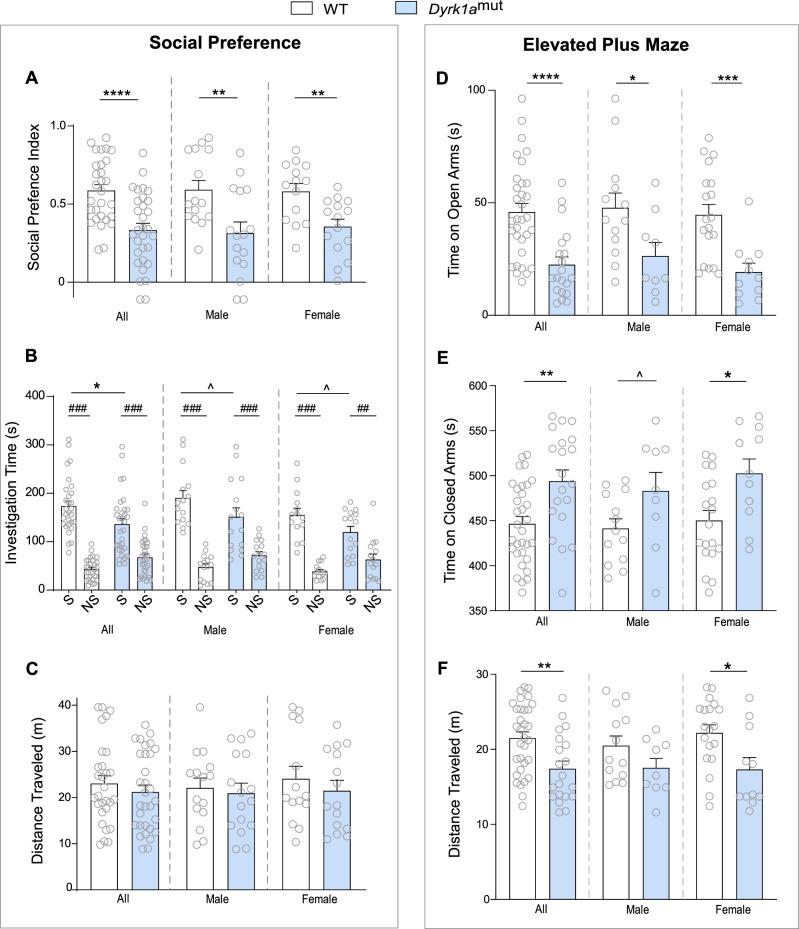
*Dyrk1a*^mut^ mice exhibited social deficits and increased anxiety. Bar graphs of social preference index (**A**), interaction time with social (S) or non-social (NS) stimulus (**B**) and total distance traveled (**C**) of WT and *Dyrk1a*^*mut*^ mice in social preference tests. Bar graphs of time on open arm time (**D**), closed arm (**E**) and total distance traveled (**F**) of WT and *Dyrk1a*^*mut*^ mice in elevated plus maze tests. All data are shown as mean ± SEM, ^*p* < 0.2, *p < 0.05, **p < 0.01, ***p < 0.001, unpaired *t*-test (**A**, **C**–**F**), ##*p* < 0.01, ###*p* < 0.001, two-way ANOVA with multiple comparisons (**B**).

As Dyrk1a haploinsufficiency has been implicated in autism, we examined several autism-related behaviors in *Dyrk1a*^mut^ mice. We first conducted the three-chamber social preference test to assess sociability. As shown in Fig. 3A, the social preference index was significantly decreased in *Dyrk1a*^*mut*^ mice of either sex, compared to WT counterparts (WT: *n* = 29, 15M, 14F, *Dyrk1a*^*mut*^: *n* = 31, 16M, 15F, all: *p* < 0.0001; male: *p* = 0.0056; female: *p* = 0.003, unpaired *t*-test), and *Dyrk1a*^mut^ mice generally spent less time interacting with the social stimulus than WT (Fig. 3B, all: 21.5% reduction, *p* = 0.017; male: 20.5% reduction, *p* = 0.18; female: 22.8% reduction, *p* = 0.11, two-way ANOVA with multiple comparisons). No significant differences were observed in the total travel distance between WT and *Dyrk1a*^mut^ mice (Fig. 3C). These findings indicate that Dyrk1A deficiency impairs social preference in both males and females.

Additionally, we assessed anxiety-like behaviors using the elevated plus maze test. As shown in Fig. 3D, E, both male and female *Dyrk1a*^mut^ mice spent significantly less time exploring the open arms and more time staying in the closed arms, compared to WT counterparts (WT: *n* = 32, 13M, 19F, *Dyrk1a*^*mut*^: *n* = 20, 9M, 11F; all: *p* < 0.001 (open), *p* = 0.0015 (closed); male: *p* = 0.03 (open), *p* = 0.06 (closed); female: *p* = 0.0007 (open), *p* = 0.01 (closed), unpaired *t*-test). Unlike WT mice who stayed at open arms to explore, *Dyrk1a*^mut^ mice usually only reached the open arm area proximal to the center zone or ran across the open arms without any stops and returned back to the center very soon. This led to the reduced total travel distance in *Dyrk1a*^mut^ mice (Fig. 3F, all: *p* = 0.003, male: *p* = 0.13, female: *p* = 0.013, unpaired *t*-test). These findings suggest that Dyrk1A deficiency elevates anxiety in both males and females.

### LSD1 inhibitor alleviates synaptic deficits in PFC pyramidal neurons from *Dyrk1a*^mut^ mice

**Fig. 4:**
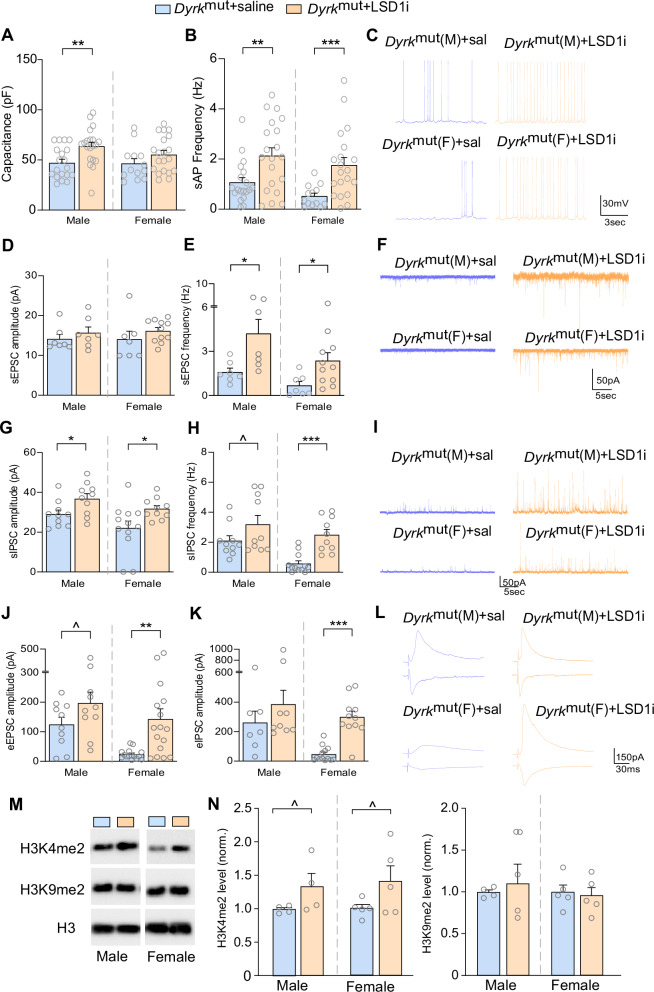
LSD1 inhibition rescues synaptic deficits in PFC pyramidal neurons of *Dyrk1a*^mut^ mice. **A** Bar graphs of membrane capacitance of PFC pyramidal neurons from male and female *Dyrk1a*^mut^ mice treated with saline or an LSD1 inhibitor, GSK-LSD1 (5 mg/kg, i.p., once daily for 3 days). **B** Bar graphs of spontaneous action potential (sAP) frequency of PFC pyramidal neurons from male and female *Dyrk1a*^mut^ mice treated with saline or GSK-LSD1. **C** Representative sAP traces. Bar graphs of sEPSC amplitude (**D**), sEPSC frequency (**E**), sIPSC amplitude (**G**), or sIPSC frequency (**H**) in PFC pyramidal neurons from male and female *Dyrk1a*^mut^ mice treated with saline or GSK-LSD1. Representative sEPSC (**F**) or sIPSC (**I**) traces. Bar graphs of eEPSC amplitude (**J**) or eIPSC amplitude (**K**) in PFC pyramidal neurons from male and female *Dyrk1a*^mut^ mice treated with saline or GSK-LSD1. **L** Representative eEPSC and eIPSC traces. Representative immunoblots (**M**) and quantitation (**N**) of H3K4me2 and H3K9me2 protein levels in the nuclear fraction of PFC from male and female *Dyrk1a*^mut^ mice treated with saline or GSK-LSD1. All data are shown as mean ± SEM, ^*p* < 0.2, *p < 0.05, **p < 0.01, ***p < 0.001, un*p*aired *t*-test.

To find out a therapeutic avenue to rescue the phenotypes of *Dyrk1a*^mut^ mice, we applied the LSD1 inhibitor, GSK-LSD1 (5 mg/kg, i.p., once daily for 3 days), followed by electrophysiological recordings of PFC pyramidal neurons from *Dyrk1a*^mut^ mice. We found that membrane capacitance was slightly increased in *Dyrk1a*^mut^ mice (Fig. 4A). The frequency of synaptic-driven spontaneous action potentials (sAP) was significantly elevated in PFC pyramidal neurons from both male and female *Dyrk1a*^mut^ mice treated with GSK-LSD1 (Fig. 4B, C, *n* = 13–20 cells/2–4 mice of each sex per group; male: *t*_31_ = 2.9, *p* = 0.007; female: *t*_24_ = 3.8, *p* = 0.0008, *t*-test).

In addition, sEPSC frequency, but not amplitude, was significantly enhanced in both sexes of *Dyrk1a*^mut^ mice (Fig. 4D–F, *n* = 7–11 cells/3–4 mice of each sex per group; sEPSC amplitude, males: *t*_11_ = 0.8, *p* = 0.42; females: *t*_8_ = 0.9, *p* = 0.37; sEPSC frequency, males: *t*_7_ = 2.6, *p* = 0.033; females: *t*_14_ = 2.8, *p* = 0.015, *t*-test). Furthermore, sIPSC amplitude and frequency were strongly elevated by LSD1 inhibition in *Dyrk1a*^mut^ mice (Fig. 4G–I, *n* = 10–12 cells/3–4 mice of each sex per group; sIPSC amplitude, male: *t*_17_ = 2.4, *p* = 0.03; female: *t*_16_ = 2.5, *p* = 0.026; sIPSC frequency, male: *t*_14_ = 1.6, *p* = 0.13; female: *t*_14_ = 4.7, *p* = 0.0004, *t*-test). LSD1 inhibitor also significantly elevated the amplitude of eEPSC and eIPSC in *Dyrk1a*^mut^ mice, and the rescuing effect was particularly prominent in *Dyrk1a*^mut^ females (Fig. 4J–L, eEPSC, *n* = 10–16 cells/3–4 mice of each sex per group; male: *t*_16_ = 1.6, *p* = 0.12; female: *t*_15_ = 3.4, *p* = 0.0039; eIPSC, *n* = 7–13 cells/3–4 mice of each sex per group; male: *t*_14_ = 1.0, *p* = 0.33; female: *t*_13_ = 5.9, *p* < 0.0001, *t*-test). Collectively, these results indicate that LSD1 inhibitor significantly ameliorated synaptic deficits in PFC neurons from *Dyrk1a*^mut^ mice.

To find out potential reasons underlying this synaptic rescue, we performed Western blotting experiments to examine the effect of LSD1 inhibitor on histone marks linked to gene activation or repression. We found that systemic administration of GSK-LSD1 (5 mg/kg, i.p., once daily for 3 days) increased the level of permissive H3K4me2, but not repressive H3K9me2, in cortical nuclear fraction of *Dyrk1a*^mut^ mice (Fig. 4M, N, *n* = 9 (4M, 5F) per group; H3K4me2, male: *p* = 0.19; female: *p* = 0.14; H3K9me2, male: *p* = 0.7; female: *p* = 0.7, Welch’s *t* test). The LSD1 inhibitor-induced elevation of H3K4me2 could lead to synaptic gene upregulation, contributing to the elevation of synaptic function in PFC neurons of *Dyrk1a*^mut^ mice.

### LSD1 inhibitor ameliorates behavioral deficits of *Dyrk1a*^mut^ mice

**Fig. 5:**
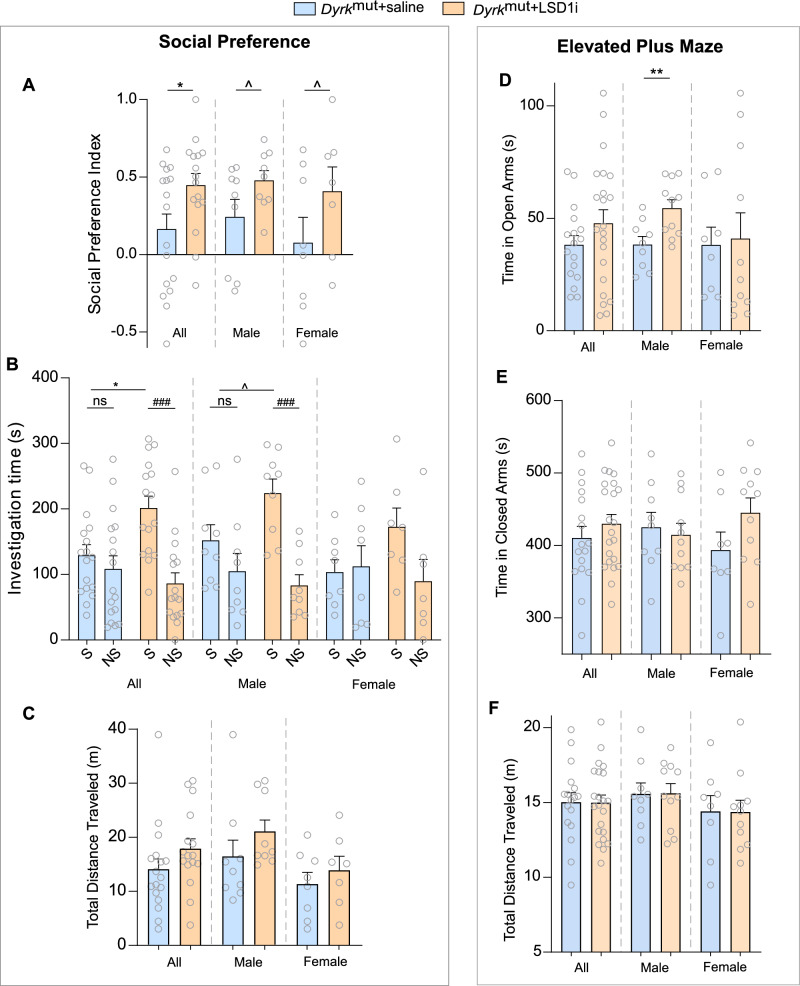
LSD1 inhibition mitigates behavioral abnormalities of *Dyrk1a*^mut^ mice. Bar graphs of social preference index (**A**), interaction time with social (S) or non-social (NS) stimulus (**B**) and total distance traveled (**C**) of *Dyrk1a*^*mut*^ mice treated with GSK-LSD1 or saline in social preference tests. Bar graphs of time spent in open arms (**D**), closed arms (**E**), and total distance traveled (**F**) of *Dyrk1a*^*mut*^ mice treated with GSK-LSD1 or saline in elevated plus maze tests. All data are shown as mean ± SEM, ^*p* < 0.2, *p < 0.05, **p < 0.01, ***p < 0.001, unpaired *t*-test or M-W test (**A**, **C**–**F**), ###*p* < 0.001, two-way ANOVA with multiple comparisons (**B**).

Next, we examined the therapeutic effect of LSD1 inhibitor on behavioral abnormalities in *Dyrk1a*^mut^ mice. Animals were given GSK-LSD1 (5 mg/kg, i.p., once daily for 3 days) or saline control, followed by elevated plus maze tests and social preference tests in the following two days. We found that GSK-LSD1-treated *Dyrk1a*^mut^ mice (*n* = 16, 9M, 7F), had significantly higher social preference indexes than saline-treated *Dyrk1a*^mut^ mice (*n* = 17, 9M, 8F) (Fig. 5A, all: *p* = 0.028; male: *p* = 0.16; female: *p* = 0.17, unpaired *t*-test or Mann–Whitney test). GSK-LSD1-treated *Dyrk1a*^mut^ mice spent more time interacting with social stimuli than non-social stimuli (Fig. 5B, all: *p* < 0.001; male: *p* < 0.001, female: *p* = 0.11, two-way ANOVA with multiple comparison), while saline-treated *Dyrk1a*^mut^ mice did not show much preference among the two stimuli. Significantly more social time was found with GSK-LSD1 treatment in the pooled data (Fig. 5B, all: 55.8% increase, *p* = 0.028; male: 47.2% increase, *p* = 0.13; female: 66.4% increase, *p* = 0.34, two-way ANOVA with multiple comparison). No significant change in locomotion was found with GSK-LSD1 treatment (Fig. 5C).

In elevated plus maze test, we found that GSK-LSD1-treated *Dyrk1a*^mut^ mice (*n* = 17, 9 M, 8 F) spent more time on open arms than saline-treated *Dyrk1a*^mut^ mice (*n* = 22, 11 M, 11 F), and the improvement was significant in male mice (Fig. 5D, all: *p* = 0.23, male: *p* = 0.006, female: *p* = 0.85, unpaired *t*-test). No significant change in time on closed arms was found with GSK-LSD1 treatment (Fig. 5E). Locomotion was also unchanged (Fig. 5F). Taken together, these results indicate that LSD1 inhibitor significantly mitigated behavioral deficits in *Dyrk1a*^mut^ mice.

## Discussion

Overexpression or haploinsufficiency of *Dyrk1a* has been causally linked to Down Syndrome or autism [15, 16]. Early immunoblotting analyses found that *Dyrk1a* overexpression affected pathways involved in synaptogenesis and synaptic plasticity [13]. Using unbiased transcriptomic analysis, we found that *Dyrk1a* mutant mice of each sex had the downregulation of genes enriched in synaptic transmission and modulation in frontal cortex, including GABA synthesis enzymes, GABA transporters, GABAergic neuronal markers, glutamate transporters, glutamate receptors, and serotonin receptors. Similar to our RNA-seq data, proteomic studies found the deregulation of pre-synaptic proteins in hippocampus by decreased *Dyrk1a* gene dosage in postnatal glutamatergic neurons [14]. Multiplexed imaging studies also identified the reduction of synaptic F-actin and other synaptic proteins (Bassoon and Homer1a) by RNAi knockdown of *Dyrk1a* [22].

Corroborated with the transcriptomic results, our electrophysiological studies found the marked reduction of cortical glutamatergic and GABAergic synaptic function by *Dyrk1a* mutation, as indicated by the significantly smaller spontaneous and evoked EPSC and IPSC in PFC pyramidal neurons of male and female *Dyrk1a*^*mut*^ mice. Similarly, decreased miniature IPSC was found in CA2 and CA3 hippocampal pyramidal neurons of *Dyrk1a*^*+/−*^ mice, suggesting the important role of *Dyrk1a* in GABAergic inhibition [22]. *Dyrk1a* knockin mice carrying an autism mutation (I48K) had decreased miniature EPSC but unchanged miniature IPSC in CA1 hippocampal pyramidal neurons [24]. These physiological alterations could directly contribute to E/I imbalance caused by *Dyrk1a* haploinsufficiency [13, 22].

Correlated with our electrophysiological alterations were the manifestation of autism-like social deficits and elevated anxiety in *Dyrk1a*^*mut*^ mice. Defective social interactions were also found in *Dyrk1a*^+/−^ mice [18, 25]. Other behavioral abnormalities associated with *Dyrk1a* gene dosage include cognitive deficits and epileptic activity [14, 18, 25].

One key issue is to find a viable therapeutic avenue for DYRK1A-linked diseases. Several DYRK1A inhibitors have been developed to attenuate Down Syndrome associated with DYRK1A overexpression [48]. Treating autism-like syndromes linked to DYRK1A haploinsufficiency is more challenging [49]. Neonatal (P0–P7) IGF-1 injection rescued microcephaly and cellular undergrowth in conditional heterozygous *Dyrk1a* knockout mice [19], suggesting that growth factor signaling could be targeted for early intervention. Another study with early chronic lithium treatment via mother’s milk (P0–P21) of *Dyrk1a* knockin mice also found the long-lasting rescue of neuronal, synaptic and behavioral phenotypes [24]. While promising, these treatments are practically unfeasible before the diagnosis of diseases based on manifested phenotypes.

Large-scale sequencing has revealed that the most prominent autism risk genes encode proteins for synaptic formation, transcriptional regulation and chromatin remodeling pathways [16, 17]. It prompted the idea that targeting epigenetic enzymes to normalize the transcription of synaptic genes could be a convergent intervention strategy for autism [33]. Indeed, pharmacological agents for histone modifiers, such as histone deacetylases (HDAC) and euchromatic histone methyltransferases (EHMT) inhibitors, have been shown to produce effective rescue in various autism models [36, 38, 45, 50].

Here, we found that a short treatment of adult *Dyrk*^*mut*^ mice with a specific inhibitor of lysine-specific histone demethylase 1A (LSD1) led to the significant rescue of excitatory and inhibitory synaptic function in PFC, as well as the mitigation of social preference deficits (both sexes) and anxiety-related behavior (males only). Consistently, LSD1 was perceived as a negative modulator of the glutamatergic synapse because increasing LSD1 activity dampened glutamatergic transmission [51]. In agreement with the therapeutic effects of LSD1 inhibitor in *Dyrk*^*mut*^ mice, it also restored NMDAR-mediated synaptic currents and social deficits of male *Shank3*-deficient mice [32]. Moreover, LSD1 inhibitors restored epigenetic machinery and gene expression, as well as improved autism symptoms, in other neurodevelopmental disorder models [34, 37, 41].

How could an LSD1 inhibitor rescue synaptic currents in *Dyrk*^*mut*^ mice? LSD1 acts as a histone demethylase to interact with histone H3K4/H3K9/H3K20 to regulate gene expression [52–54]. One possibility is that LSD1 inhibition increases the level of permissive H3K4me2 or decreases the level of repressive H3K9me2 on synaptic gene promoters, leading to their elevation in *Dyrk*^mut^ mice. We observed the elevation of H3K4me2 level in PFC by systemic administrations of an LSD1 inhibitor. However, the detailed molecular mechanisms underlying the rescuing effects of LSD1 inhibitor in male and female *Dyrk*^mut^ mice await to be further investigated.

DYRK1A plays a pivotal role in autism and other neurodevelopmental diseases [1, 55]. The current and other preclinical studies have demonstrated the therapeutic potential of LSD1 inhibitors on autism models [32, 37, 41], setting the scientific foundation for targeting LSD1 in clinical trials for autism.

## Supplementary information

Supplementary Tables (excel file)

## Data Availability

RNAseq data will be deposited to the public repository Gene Expression Omnibus (GEO). All other data are available upon request.
